# Encapsulation of *Tenebrio molitor* Hydrolysate with DPP-IV Inhibitory Activity by Electrospraying and Spray-Drying

**DOI:** 10.3390/nano14100840

**Published:** 2024-05-10

**Authors:** Carmen Berraquero-García, Lydia Martínez-Sánchez, Emilia M. Guadix, Pedro J. García-Moreno

**Affiliations:** Department of Chemical Engineering, Faculty of Sciences, University of Granada, 18071 Granada, Spain; lidilms99@correo.ugr.es (L.M.-S.); eguadix@ugr.es (E.M.G.)

**Keywords:** antidiabetic peptides, enzymatic hydrolysis, nano-microencapsulation, electrospraying, spray-drying

## Abstract

This study investigates the encapsulation of *Tenebrio molitor* hydrolysate exhibiting DPP-IV inhibitory activity by spray-drying and electrospraying techniques. First, we optimized the feed formulation and processing conditions required to obtain nano-microcapsules by electrospraying when using Arabic gum as an encapsulating agent and pullulan and Tween 20 as additives. The optimum formulation was also dried by spray-drying, where the removal of the additives was also assayed. Morphology analysis reveals that electrosprayed capsules have a smaller size (1.2 ± 0.5 µm vs. 12.4 ± 8.7 µm) and greater uniformity compared to those obtained by spray-drying. Regarding the surface nitrogen content and DPP-IV inhibitory activity, our results show no significant difference between the electrosprayed capsules and spray-dried capsules containing additives (IC_50_ of ~1.5 mg protein/mL). Therefore, it was concluded that adding additives during spray-drying allows for a similar encapsulation efficiency and reduced degradation during processing, as achieved by electrospraying technique but providing higher productivity. On the other hand, spray-dried capsules without additives displayed a higher surface nitrogen content percentage, which was mainly due to the absence of Tween 20 in the feed formulation. Consequently, these capsules presented a higher IC_50_ value (IC_50_ of 1.99 ± 0.03 mg protein/mL) due to the potential degradation of surface-exposed peptides.

## 1. Introduction

According to the International Diabetes Federation, 537 million people have diabetes, with type 2 diabetes mellitus (T2DM) being the most common, accounting for around 90% of all cases. This number is expected to increase to 643 million by 2030 [1]. T2DM is a metabolic disorder characterized by chronically high blood sugar levels due to insulin resistance and insufficient insulin secretion [2]. Insulin administration remains the primary treatment available; however, subcutaneous injection, which is undesirable for patients, is required as oral administration is impractical [3]. Hence, there is an increased interest in the development of alternative therapies. In particular, the inhibition of the enzyme dipeptidyl-peptidase IV (DPP-IV) has become a promising approach for controlling glycemic levels [4], leading to the development of oral antihyperglycemic drugs known as gliptins [5,6]. However, these drugs present significant risks [7], and increasing efforts have been focused on the use of bioactive peptides due to their potential to serve as antidiabetic agents without exhibiting side effects [8].

Bioactive peptides are mainly derived from the enzymatic hydrolysis of animal proteins (i.e., milk, fish, and others) [9,10,11]. Yet, the increasing the global population requires novel sustainable protein sources to meet higher demands while minimizing environmental effects [12]. Insects have emerged as a new sustainable protein source in recent years [13]. Particularly, *Tenebrio molitor* larvae (mealworms) have been recognized as a novel food by the European Food Safety Agency and are known for their high nutritional value in terms of protein and fat content [14,15], digestibility [16,17], and functional ability [18,19]. Additionally, several studies have highlighted the capacity of *T. molitor* to produce DPP-IV inhibitors through enzymatic hydrolysis [12,20].

The oral administration of bioactive peptides, for instance, in the form of functional food, is a simple and convenient approach for patients to counteract T2DM. However, biopeptides present some drawbacks that need to be addressed: high hygroscopicity leading to instability and decreased bioactivity [21], bitterness from hydrophobic amino acid residues [22], the low water solubility of highly hydrophobic peptides hindering incorporation into food formulation [23], physicochemical instability during storage and digestion [24], and limited bioaccessibility in the intestine [25]. A common strategy employed to overcome these drawbacks is the encapsulation of the protein hydrolysates/peptides used as food bioactive ingredients.

Encapsulation consists of the entrapment of the peptides within a matrix of one or more encapsulating agents [26]. Despite extensive research on the encapsulation of various substances such as lipids (e.g., omega-3 fatty acids) [27], vitamins [28], polyphenols [29], and other drugs [30,31], there have been relatively fewer studies conducted on the encapsulation of bioactive peptides. Furthermore, most of the research on encapsulating bioactive peptides has employed methods like coacervation, freeze-drying, and spray-drying [32]. While coacervation offers benefits such as high efficiency for flavorings and protection against oxidation at mild temperature conditions with potential for controlled release, it also has limited applications in the food industry due to its sensitivity to pH and ionic strength between the wall and core material [33]. Freeze drying can ensure physicochemical and bioactive stability but requires a significant amount of time–labor-cost resources compared to previous findings that favor spray-drying [34]. Spray-drying is widely used in the food industry for encapsulating bioactive components because of its versatility in terms of solvents and encapsulating agents, as well as its cost-effective benefits and high productivity [35]. This technique is based on the dehydration of finely atomized droplets due to their contact with a hot gas, such as air or, less commonly, an inert gas (e.g., nitrogen), resulting in dry particles [36,37]. The encapsulation of bioactive protein hydrolysates/peptides with antidiabetic activity by this technique has been investigated in various studies using diverse encapsulating agents, such as trehalose, mannitol and sorbitol [38], and maltodextrin combined with gum Arabic [39] and with agar and carrageenan [40]. Nevertheless, the digestion of trehalose and maltodextrin releases glucose, which is not desirable when producing functional food with antidiabetic activity, and carrageenan has been linked to glucose intolerance [41,42]; thus, alternative encapsulating agents should be used instead. In this regard, Arabic gum is a polysaccharide that is frequently used as an encapsulating agent due to its high solubility in water, leading to a low viscous solution, as well as its stabilizing and emulsifying properties [43]. Furthermore, Arabic gum does not provide glucose during digestion as it is not mostly digestible, making it suitable for encapsulating antidiabetic peptides [44]. In addition, studies have shown that Arabic gum may lower blood glucose levels by inhibiting the absorption of glucose in the intestine [45].

On the other hand, electrospraying is an alternative drying technique [46] consisting in the application of a high-voltage electric field between the tip of a needle and a grounded collector to induce the ejection of the solution. Upon reaching a high electrostatic field, the meniscus interface polarizes and forms a conical shape known as a Taylor cone. With increasing voltage, the electric force overcomes the surface tension, and a jet is released towards the collector [47]. Under sufficiently low solution viscoelasticity conditions, the jet destabilizes to form small, charged droplets that disperse due to electrostatic forces [48]. Meanwhile, solvent evaporation occurs in the travel of the droplets to the collector, leading to the obtaining of nano-microcapsules in powdered form [49]. Although this method has been previously used for the encapsulation of diverse bioactive protein hydrolysates/peptides [50,51], to the best of our knowledge, there are no previous studies on the encapsulation of peptides exhibiting antidiabetic activity.

Therefore, this study aimed to investigate encapsulation by electrospraying a *Tenebrio molitor* protein hydrolysate containing bioactive peptides which exhibit antidiabetic activity. For the sake of comparison between encapsulation technologies, the encapsulation of the hydrolysate by spray-drying was also evaluated. Thus, this work specifically examines the efficacy of electrospraying and spray-drying techniques in protecting these bioactive peptides. Initially, the formulation of the feed solution was optimized to obtain a stable electrospraying process and encapsulates with adequate morphology. Subsequently, encapsulates where produced using the optimal formulation by electrospraying and also by spray-drying. The obtained encapsulates were characterized based on their morphology and particle size distribution. Moreover, their surface nitrogen content was evaluated to determine the encapsulation efficiency. Finally, the *in vitro* retention of DPP-IV-inhibitory activity by the encapsulated hydrolysate was investigated.

## 2. Materials and Methods

### 2.1. Materials

Whey protein hydrolysate (84 wt.% protein content), which was used as model protein in the optimization of the formulation, and the Arabic gum were kindly donated by Abbott Laboratories S.A. (Granada, Spain) and Nexira (Serqueux, France), respectively. Pullulan was supplied by Hayashibara Co., Ltd. (Okayama, Japan). Tween 20 was purchased from Sigma Aldrich (Darmstadt, Germany). *Tenebrio molitor* meal (68.01 wt.% protein) was kindly provided by Tebrio (Salamanca, Spain), which was ground to powder. Alcalase (subtilisin, EC 3.4.21.62) was purchased from Novozymes (Bagsvaerd, Denmark). DPP-IV enzyme (EC 3.4.14.5) and the substrate Gly-pro-p-nitroanilide were supplied by Sigma Aldrich (St. Louis, MO, USA) and stored at −20 °C until use.

### 2.2. Production of the Tenebrio molitor Protein Hydrolysate

The enzymatic hydrolysis of *Tenebrio molitor* meal was conducted in a jacketed reactor connected to an automatic titrator (718 Stat Titrino, Metrohm, Herisau, Switzerland). Briefly, the *T. molitor* hydrolysis was conducted at 50 °C and pH 8. Thirty g/L protein was dissolved in distilled water and Alcalase 2.4 L (EC 3.4.21.62) was added at the beginning of the reaction at a 3% enzyme-to-substrate (protein) ratio. The reaction continued until the degree of hydrolysis (DH), measured by the pH-stat method [52], was 20%. The resulting hydrolysate was then deactivated by heating the solution at 100 °C for 15 min, centrifuged at 5300× *g* for 15 min, and vacuum-filtered through an 8 µm cellulose filter. The supernatant was lyophilized (LyoMicron, Coolvacuum Technologies S.L., Barcelona, Spain) and the powdered product was stored at −20 °C. The nitrogen content of the obtained hydrolysate powder was determined in triplicate according to the Dumas method using a Flash 2000 CHNS/O elemental analyzer (Thermo Scientific, Waltham, MA, USA). Protein content was calculated assuming a nitrogen-to-protein factor of 5.6 [53], resulting in 68.47 ± 0.39 wt.%.

### 2.3. Production of Electrosprayed Capsules

To encapsulate the *T. molitor* hydrolysate, Arabic gum and pullulan were used as encapsulating material and Tween 20 was employed as surfactant. These compounds were dissolved in distilled water and stirred overnight (350 rpm) at room temperature. The concentration of protein was kept at 20 wt.% in the final capsule, whereas Arabic gum at 15 wt.% and pullulan at 1–4 wt.% were referred to the feed formulation. Tween 20 at 1 wt.% (referred to Arabic gum and pullulan) was also used. The solution was electrosprayed utilizing a system comprising a drying chamber, equipped with a high-voltage power supply (adjustable up to 30 kV), a syringe pump, and a collector plate (15 × 15 cm, made of stainless steel) (SpinBox Electrospinning; Bioinicia, Valencia, Spain). A 5 mL syringe containing the solutions was mounted onto the syringe pump and 16 G needles (Proto Advantage, Hamilton, ON, Canada) were used. A monoaxial single-phase emitter (one needle) was used for the optimization of the formulation, while a monoaxial multi-emitter consisting of five parallel needles was used for increasing the productivity of the optimum formulation. The emitter was positioned 15 cm away from the collector plate in a horizontal configuration. The flow rate and voltage were kept at 0.2 mL/h and 22 kV, respectively. The electrospraying process was carried out at room temperature and relative humidity conditions (21–27 °C, 36–51 %RH) in batches of 1 h. The powder collected from the different batches was gently mixed and stored in plastic Eppendorf tubes at 4 °C until further use.

### 2.4. Production of the Spray-Dried Capsules

The obtained optimum feed solution containing the *T. molitor* solution was prepared as previously described to produce electrosprayed capsules, with and without pullulan and Tween 20. The spray-drying process was carried out in a laboratory-scale spray-drier (Büchi B-190; Büchi Labortechnik, Flawill, Switzerland) using a nozzle with a diameter of 0.7 mm (Büchi, Flawill, Switzerland). The temperature of inlet air was set at 190 °C and the temperature of the outlet air was kept at 95–97 °C. The drying airflow was fixed at 25 Nm^3^/h. Once the different microcapsules were collected, they were stored at −20 °C in the dark until analysis.

### 2.5. Characterization of the Capsules

#### 2.5.1. Morphology and Particle Size Distribution

The morphology of the capsules was examined using scanning electron microscopy (SEM) on a FESEM microscope (LEO 1500 GEMINI, Zeiss, Germany). Depending on the employed encapsulation technique, a slender layer of microcapsules (via spray-drying) or a segment of aluminum foil measuring approximately 0.5 × 0.5 cm enclosing the sample (via electrospraying) was affixed onto a carbon tape on a pin and carbon-coated using an EMITECH K975X Turbo-Pumped Thermal Evaporator (Quorum Technologies, Lewes, UK). SEM images were captured in the range of 2K×–20K× in case of the electrosprayed capsules, and 200×–2K× in the case of spray-dried capsules with a 3 kV accelerating voltage and 30 µm aperture. The particle size distributions and mean diameters were determined by measuring 200 randomly selected particles using the ImageJ software (https://imagej.net/ij/index.html, accessed on 7 May 2024) (National Institute of Health, Bethesda, MD, USA).

#### 2.5.2. X-ray Photoelectron Spectroscopy (XPS)

The presence of *T. molitor* hydrolysate on the surface of the microparticles was analyzed by determining the nitrogen surface using X-ray photoelectron spectroscopy (XPS). The microparticles were transferred to a glass slide and analyzed using a Kratos Axis Ultra-DLD (Kratos Analytical, Manchester, UK) The samples were subjected to both an overall spectrum analysis (under the conditions of 75 W power and 160 eV pass energy) and a quantification of carbon, oxygen, and nitrogen. Charge neutralization was activated, and the penetration depth was maintained at less than 10 nm.

#### 2.5.3. DPP-IV Inhibitory Activity

The DPP-IV-inhibitory activity of the produced *T. molitor* hydrolysate and the microcapsules loaded with the hydrolysate was measured following a modified protocol based on Lacroix and Li-Chan (2012) [54]. Briefly, 25 μL of DPP-IV enzyme at 0.02 U/mL were incubated at 37 °C with 100 μL of *T. molitor* hydrolysate or capsules aqueous solutions at varying concentrations (0.25–5 mg/mL) for 10 min. Subsequently, the reaction was initiated by the addition of 50 μL of 1 mM Gly-Pro-p-nitroanilide. The release of the reaction product (p-nitroanilide) was monitored by measuring absorbance at 405 nm every 2 min for 2 h at 37 °C using a Multiskan FC microplate photometer (Thermo Scientific, Vantaa, Finland). Each sample was analyzed in triplicate and a color control was added. The inhibition activity was calculated by comparing the reaction progress to a control (distilled water) as follows:

(1) $$DPP-IV\;inhibition\;(\%)=\frac{\left(1-p_{i}\right)}{p_{0}}\cdot 100$$

where *p_i_* is the slope in the presence of the inhibitor (peptide) and *p*_0_ is the slope obtained in the absence of the inhibitor (control). The half-maximal inhibitory concentration (IC_50_) of each sample was calculated. Results are expressed in mg protein/mL as the mean ± standard deviation.

#### 2.5.4. Statistical Analysis

The dataset underwent analysis of variance (ANOVA) using Statgraphics version 5.1 (Statistical Graphics Corp., Rockville, MD, USA). Tukey’s honest significant difference (HSD) multiple range test was employed at the 95% confidence level (*p* < 0.05) to discern significant variations among mean values.

## 3. Results

### 3.1. Optimization of the Formulation for Electrospraying

Arabic gum was employed as the main encapsulating agent due to its excellent encapsulating properties such as water solubility, stabilizing and emulsifying properties, and the low viscosity of its aqueous solutions [43]. Additionally, Tween 20, a non-ionic surfactant, was used to improve the solution properties by reducing surface tension and enhancing the viscoelastic properties [55,56]. Finally, to enhance the stability of the Taylor cone and, thus, the electrospraying process, the addition of pullulan as a secondary encapsulating agent was tested. The addition of pullulan leads to an increase in the viscoelasticity of the feed solution due to the interactions and entanglements that occur between the pullulan chains and between the chains of Arabic gum and pullulan [49]. The latter enhances the stability of the Taylor cone when working at a higher flowrate, which increases productivity [57]. Nevertheless, pullulan exhibits a high electrospinning capacity in water-based solutions, which is not beneficial for the intended application, as electrospun fibers, unlike nano/microcapsules, result in continuous mats that are challenging to disperse in any food matrix [58].

**Figure 1:**
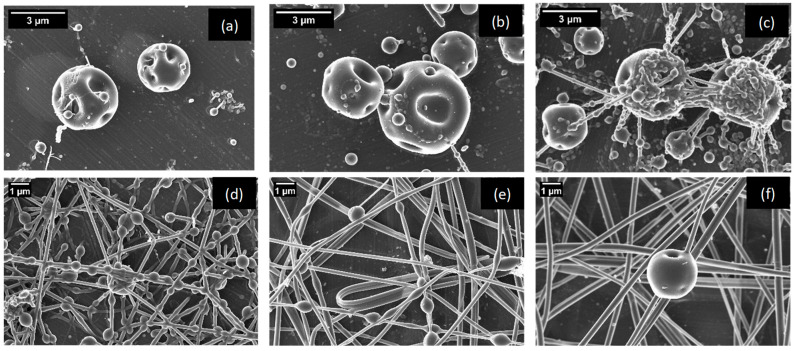
Morphology of the capsules obtained when using 20 wt.% whey protein (in the final capsule), 15 wt.% Arabic gum, 1 wt.% Tween 20 (with respect to the total biopolymers), and pullulan 0.5 wt.% (**a**), 0.75 wt.% (**b**), 1 wt.% (**c**), 2 wt.% (**d**), 3 wt.% (**e**) and 4 wt.% (**f**).

First, the optimization of the Arabic gum content in the feed was investigated. Since Arabic gum in the range 15–40 wt.% does not properly electrospray, and based on previous studies using glucose syrup and dextran [57], we fixed the Arabic gum concentration in the feed to 15 wt.%, which will lead to low viscoelasticity and a reduced capsule size, and adding a low content of pullulan and 1 wt.% of Tween 20 (with respect to total biopolymer). Therefore, the solution comprised 20 wt.% (in the final capsule) of whey protein, which was used as model protein, 15 wt.% Arabic gum and 1 wt.% Tween 20 (referred to pullulan and Arabic gum concentrations) was electrosprayed, varying the concentration of pullulan in the range 0.5–4 wt.%. Samples were characterized using SEM (Figure 1). As expected, in capsules with lower amounts of pullulan (i.e., 0.5, 0.75, and 1 wt.%), there was only a faint hint of any strands emerging from the capsules, while for concentrations of above 1 wt.%, a greater number and longer strands could be observed. When pullulan concentrations reached 2 wt.%, a notable change in the morphology of the capsules became apparent, transitioning from spheres to interwoven and thick fibers, which turned especially visible at 3 and 4 wt.%. This was attributed, as mentioned before, to the high electrospinning capability exhibited by pullulan [58]. It should be noted that solutions with both 0.5 wt.% and 0.75 wt.% pullulan concentrations manifested a marginally superior capsule morphology in comparison to the encapsulates with a 1 wt.% concentration. Nevertheless, the observed productivity was much lower for the first two formulations, when the pullulan concentration was kept under 1 wt.%. These two formulations (those with 0.5 and 0.75 wt.%) also presented a higher instability in achieving and maintaining a stable Taylor cone during the electrospraying process, which was manifested as an elongated shape of the cone as well as the reduced productivity of the process. Taking all these aspects into consideration, the formulation composed of 1 wt.% pullulan was deemed as the optimum.

### 3.2. Morphology and Particle Size Distribution of Electrosprayed and Spray-Dried Capsules

**Figure 2:**
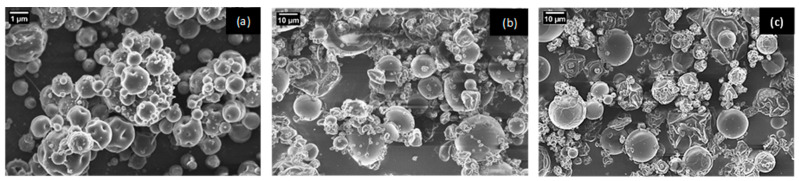
SEM images of capsules loaded with *Tenebrio molitor* hydrolysate formulated with pullulan and Tween 20, either electrosprayed (**a**) or spray-dried (**b**), and formulated without pullulan and Tween 20 and spray-dried (**c**).

SEM micrographs of the resulting *T. molitor* encapsulates from electrospraying (a) and spray-drying (b–c) processes are presented in Figure 2. The same formulation containing *T. molitor* hydrolysate, Arabic gum, Tween 20, and pullulan was both electrosprayed (Figure 2a) and spray-dried (Figure 2b). Figure 2c shows the SEM image of the capsules obtained by spray-drying, where the dried feed solution only contains Arabic gum and hydrolysate (e.g., without the additives pullulan and Tween 20). It can be observed in the three SEM images that most of the capsules were non-agglomerated, exhibiting a spherical shape with a wrinkled surface and concavities. The loss of spherical shape exhibited by various capsules might be attributed to an incomplete solvent evaporation and the unraveling of biopolymer chains, causing the unsolidified particles to be prone to deformation upon contacting the collector/walls of the equipment [59]. Regarding the wrinkles and concavities present on the surface of the capsules, these are characteristic of Arabic gum and could be attributed to the non-uniform distribution of this biopolymer within the droplets [59,60] and the slow formation of this encapsulating agent film/crust during the drying process [61]. In the case of spray-dried capsules, several authors also suggest that this phenomenon may be related to the contraction experienced by the particles during drying and cooling [62]. Comparing Figure 2b,c, it was noticeable that capsules containing pullulan and Tween 20 showed a moderately smoother surface. This reduction in wrinkles and indentations on the surface of the capsules was associated with the surfactant, owing to the preferential migration of Tween 20 molecules onto the surface of droplets/particles during atomization and drying [63,64].

**Figure 3:**
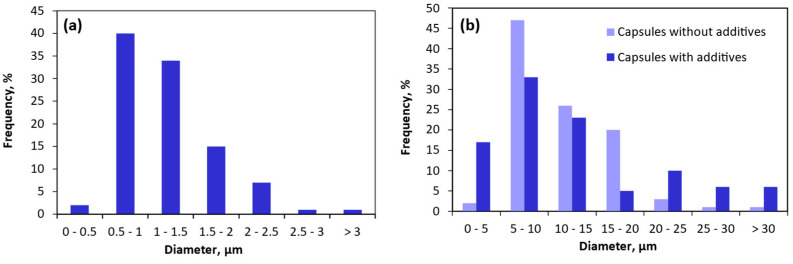
Particle size distribution of the capsules produced by electrospraying (**a**) and spray-drying (**b**).

The particle size distribution of the electrosprayed and spray-dried capsules is depicted in Figure 3. The particle size distribution of nano-microcapsules obtained by electrospraying was significantly narrower (ranging from 0.5 to 3.1 µm) compared to that of the microcapsules produced by spray-drying (ranging from 4.6–3.4 to 39–36 µm with/without the presence of additives, respectively). For an electrospraying technique, capsules with an average size of 1.2 ± 0.5 µm were produced, with 91% of them having a diameter below 2 µm. This smaller particle size obtained employing the electrospraying method is attributed to the breakup of droplets through Coulombic repulsion forces during the process, promoting the formation of ultrafine encapsulates with a narrow diameter distribution at the high voltage used (22 kV) [35]. This is also facilitated by the presence of Tween 20 as a surfactant, as it reduces the surface tension and aids in the disintegration of droplets, leading to the formation of finer and smaller particles [47,65].

Microcapsules obtained by spray-drying, which contain additives, had an average size of 12.4 ± 8.7 µm, while those without additives showed an average size of 11.3 ± 5.76 µm. It is worth noting that there was a higher number of microcapsules of larger size when pullulan and Tween 20 were present. Specifically, only 78% of the capsules were under 20 µm in the presence of additives compared to 95% when no additives were included. This was most likely due to the higher solids content and thus the increased viscosity of the solution provided when adding pullulan and Tween 20, bringing about larger droplets after atomization and, consequently, larger particles [36,66]. Despite the larger size of the spray-dried capsules compared to the electrosprayed ones, it can be considered sufficiently small, as it is in the order of tens of microns. In fact, according to previous studies, particles with sizes below 50 µm are not capable of being detected as individual entities in the human mouth [67]. Therefore, these microcapsules obtained by spray-drying could be employed to enrich food matrices without the capsules being perceptible on the taste buds. Moreover, a high surface-area-to-volume ratio (small capsules) results in a quicker release, as fluids can penetrate the particles more easily, facilitating enhanced bioactive diffusion and the accelerated degradation of the polymeric matrix [45]. Nevertheless, it should be highlighted that, through the spray-drying process, it is common to obtain a broad size range and particle size distribution for the produced encapsulates [68]. This variation could result in capsules exhibiting a diverse performance concerning the protection and administration of bioactive peptides [37,69].

### 3.3. Surface Nitrogen of the Capsules

**Figure 4:**
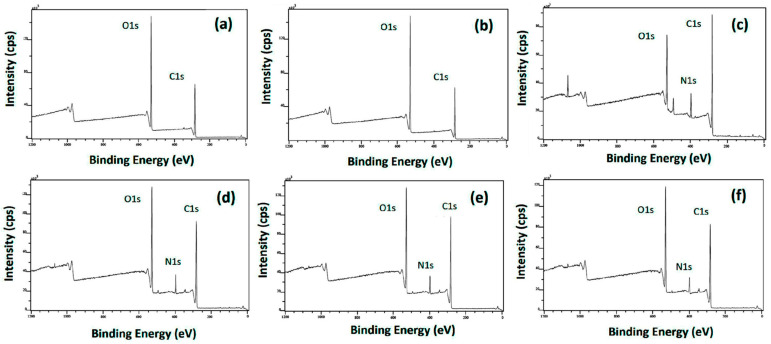
XPS spectra of Arabic gum (**a**), pullulan (**b**), *Tenebrio molitor* hydrolysate (**c**), and electrosprayed capsules (**d**) and spray-dried capsules with (**e**) and without additives (**f**).

To assess the effectiveness of the encapsulation of *T. molitor* hydrolysate, the mass concentration of nitrogen on the surface of the capsules was determined. The surface nitrogen concentration of the hydrolysate and the encapsulating agents, namely Arabic gum and pullulan, was also determined. Nevertheless, that of Tween 20, being a fluid, could not be evaluated, although nitrogen is not present in the molecule of Tween 20. The XPS spectra of the samples are illustrated in Figure 4. The various peaks correspond to the identification of carbon at 283.0 eV, nitrogen at 398.0 eV, and oxygen at 530.0 eV. While the peaks corresponding to carbon and oxygen can be observed for all the samples, the carbon peak is particularly intense for the protein hydrolysate, as expected from its composition (Figure 4c). The peak corresponding to nitrogen is only significantly present in the *Tenebrio molitor* hydrolysate (Figure 4c) and the corresponding capsules (Figure 4d–f). Therefore, the presence of nitrogen on the surface of the encapsulates indicates that some of the peptides must have migrated to the surface of the capsules during the drying processes.

**Figure 5:**
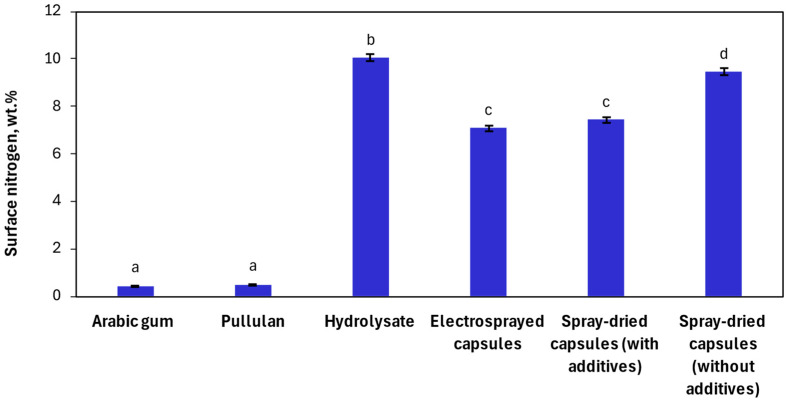
Percentage of the mass concentration of nitrogen on the surface of Arabic gum, pullulan, *Tenebrio molitor* hydrolysate, and the produced nano-microcapsules. Different letters denote significant differences between samples (*p* ≤ 0.05).

The quantification of the amount of each material at the surface of the samples is shown in Figure 5 in terms of peak intensities. As can be observed, a small concentration of nitrogen, specifically 0.44 wt.%, was found on the Arabic gum sample. This compound is primarily constituted of polysaccharides, but it also contains a small protein fraction (<2 wt.%) in its composition [70], which could justify the presence of nitrogen on its surface (small peak shown in Figure 4a). Although pullulan is a polysaccharide and, therefore, should not contain nitrogen in its composition, the XPS revealed a nitrogen concentration on its surface of 0.49 wt.%, which could be attributed to sample contamination during its preparation or measurement. Regarding the hydrolysate, a concentration of nitrogen of 10 wt.% was found when using XPS. In comparison, all capsules presented lower surface nitrogen values than the hydrolysate, which implies that encapsulation was achieved. Electrosprayed capsules exhibited 7 wt.% of surface nitrogen, spray-dried capsules containing additives had 7.4 wt.% of surface nitrogen, and spray-dried capsules without additives 9.5 wt.%. Thus, capsules produced by electrospraying and spray-drying with additives presented a similar concentration of nitrogen on the surface, whereas the highest content of surface nitrogen was obtained for the spray-dried capsules without additives. This was most likely due to the absence of surfactant, as Tween 20 molecules migrate more readily to the air–liquid interface than peptides because of their higher surface activity. Tween 20 is also capable of forming networks with the peptides and, consequently, restricting their movement to the interface [71]. Therefore, both the preferential migration and capability to form networks of Tween 20 reduce the aggregation/localization of the peptides at the interface during spray-drying and electrospraying processes. Nonetheless, it is normal to find the diffusion of the protein to the surface due to drying kinetics, as the surface activities of peptides could result in their adsorption onto the droplet surface, inducing a diffusional flux towards it. Additionally, as the evaporating droplet diminishes, its receding surface contributes to a rise in solute concentrations at the surface [72].

### 3.4. DPP-IV Inhibitory Activity

**Figure 6:**
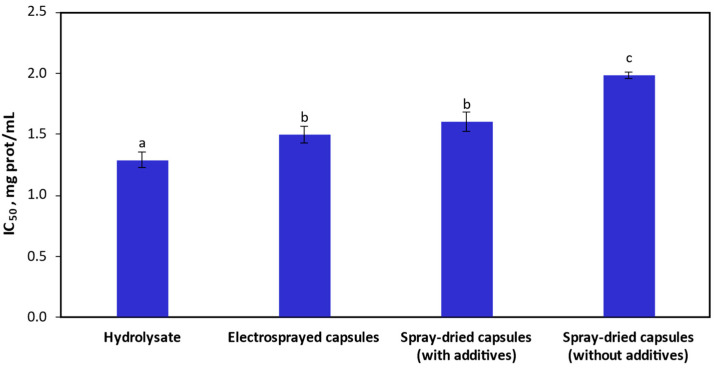
DPP-IV-inhibitory activity represented by the IC_50_ values for the *Tenebrio molitor* hydrolysate and the different encapsulates. Values are presented as the mean of three replicates ± standard deviation. Different letters denote significant differences between samples (*p* ≤ 0.05).

After encapsulation, it is crucial that the bioactive peptides comprising the hydrolysate maintain their original bioactivity. Hence, the DPP-IV-inhibitory activity of the non-encapsulated *T. molitor* hydrolysate was compared to that of the encapsulated hydrolysate. The results are expressed as the concentration of protein either in the non- or encapsulated forms at which 50% inhibition of the enzyme was achieved (IC_50_), as presented in Figure 6. The IC_50_ value for the initial *T. molitor* hydrolysate was 1.29 ± 0.07 mg protein/mL. Garzón et al. (2023) reported a similar value, approximately 1.5 mg protein/mL, for the brewer’s spent grain hydrolysate, employing Neutral protease-Purazyme and Flavourzyme enzymes for the hydrolysis procedure [40]. The inhibitory activity of the hydrolysates depends on several factors regarding their peptide composition, among which their peptide chain length (PCL), their content of hydrophobic amino acids, and the position of said amino acids play significant roles [73,74]. Particularly, short peptides (<7 amino acids), with the presence of proline between the first and fourth positions relative to N-terminal, and alanine, in the first or second position relative to the N-terminal, have been determined as favorable [75,76]. Concerning this, numerous studies indicate that *T. molitor* larvae meal exhibits a composition rich in leucine, valine, alanine, and proline [77,78]. This composition could explain the high DPP-IV inhibitory activity observed on *T. molitor* hydrolysates by various studies, reporting IC_50_ values of 0.83 and 0.91 mg protein/mL using Papain [79], and Flavourzyme [80] enzymes for hydrolysis, which aligns with our results.

In comparison, the electrosprayed encapsulates as well as the spray-dried encapsulates containing additives showed a similar percentage of DPP-IV inhibition when compared to the free hydrolysate, with an IC_50_ value of 1.50 ± 0.07 mg protein/mL 1.61 ± 0.08 mg protein/mL, respectively. On the other hand, the spray-dried capsules without additives presented a significantly higher IC_50_ value (1.99 ± 0.03 mg protein/mL) when compared to those of the electrosprayed and spray-dried (with additives) capsules. Therefore, the DPP-IV inhibitory activity decreased by 16.16%, 24.13%, and 53.60%, respectively, for the electrospraying, spray-drying with additives or spray-drying without additives in the encapsulation processes. It is worth noting that Garzón et al. (2023) reported higher values, around 3.5 mg protein/mL, for the encapsulates obtained by spray-drying using agar and/or carrageenan together with maltodextrin as carriers when encapsulating a hydrolysate with an initial IC_50_ value around 1.5 mg protein/mL [40].

Regarding the loss of bioactivity in the electrosprayed capsules, this might be attributed to a slight denaturation of the peptides due to the high voltage used or a potential detrimental interaction between the peptides and the biopolymers used. Nevertheless, the electrospraying process is conducted at room temperature, and thus, there is no significant loss of their activity. Onyekuru et al. (2021b) found that, for the encapsulation of alkaline phosphatase with PEO as an encapsulating agent, employing a voltage of 22.5 kV led to a minor loss of enzyme activity [81]. With respect to the spray-dried capsules, the decrease in the inhibitory activity of the DPP-IV enzyme was most likely due to the high temperature involved in the process, leading to the thermal degradation of the hydrolysate, although this degradation should be minimal for inlet temperatures ranging from 130 to 190 °C and outlet temperatures below 100 °C, as reported in the literature [82,83]. The loss of inhibitory activity in spray-dried capsules without additives was noticeably higher and could be related to the absence of Tween 20. This surfactant tends to migrate to the surface of the capsule and form networks with the peptides, preventing them from moving towards it and avoiding further degradation due to exposure to heat at the surface of the particle. This reduced migration of peptides to the interface also results in minimized damage caused by shear tension and dehydration stress during the drying of droplets, enhancing the bioactivity of the peptides [71]. Hence, a lower nitrogen concentration on the surface implies that peptides have remained inside the capsule, where they are better protected. In fact, after conducting a correlation comparative between the surface nitrogen and the DPP-IV enzyme inhibition, it was found that the coefficient of determination (*R*^2^) was 0.99. In addition to the process conditions and the incorporation of Tween 20, another factor that may have affected the bioactivity is the secondary encapsulating agent. No studies have been found in the literature employing pullulan as a carrier of compounds with antidiabetic activity. Nevertheless, Rahmani-Manglano et al. (2023) reported that the addition of pullulan improved the oxidative stability of omega-3 encapsulates due to its inherently impermeability to oxygen [69]. Hence, it would be interesting to investigate in future studies whether pullulan influences antidiabetic activity.

## 4. Conclusions

In this study, the encapsulation of *Tenebrio molitor* hydrolysate was studied using electrospraying and spray-drying processes with Arabic gum as the primary encapsulating agent. The addition of pullulan to improve viscoelasticity was studied, and the concentration was optimized at 1 wt.% together with the use of Tween 20 to obtain a stable electrospraying process. Electrospraying yielded better results in terms of size and particle distribution compared to spray-drying, producing smaller and more uniform capsules. There was no significant difference observed between the electrosprayed capsules and spray-dried capsules containing additives regarding the nitrogen content on their surface or DPP-IV inhibitory activity. Conversely, spray-dried capsules without additives showed a lower bioactivity and encapsulation efficiency with a higher mass concentration of nitrogen on the surface. This was attributed to the absence of surfactant, which acts to reduce the migration of peptides to the liquid/air interface during drying. Overall, it could be noted that microcapsules obtained by spray-drying when adding additives (pullulan and Tween 20) to the feed resulted in a similar encapsulation efficiency and antidiabetic activity, as achieved for the capsules obtained by electrospraying. Nevertheless, it is worth noting that spray-drying provides higher productivity than electrospraying due to its superior scalability.

## Data Availability

Data are contained within the article.
